# Plasmin-mediated proteolysis of von Willebrand factor in patients with acute and chronic liver disease

**DOI:** 10.1016/j.rpth.2025.103166

**Published:** 2025-09-01

**Authors:** Hinde El Otmani, Marilena Stamouli, Jelle Adelmeijer, William Bernal, Coen Maas, Vishal C. Patel, Ton Lisman

**Affiliations:** 1 Central Diagnostic Laboratory Research, University Medical Center Utrecht, Utrecht University, Utrecht, The Netherlands; 2 Roger Williams Institute of Liver Studies, School of Immunology & Microbial Sciences, Faculty of Life Sciences and Medicine, King’s College London, Foundation for Liver Research and King’s College Hospital, London, United Kingdom; 3 Surgical Research Laboratory, Department of Surgery, University of Groningen, University Medical Center Groningen, Groningen, The Netherlands; 4 Institute of Liver Studies & Transplantation, King's College Hospital, NHS Foundation Trust, London, United Kingdom; 5 Synapse Research Institute, Maastricht, The Netherlands; 6 Section of Hepatobiliary Surgery and Liver Transplantation, Department of Surgery, University of Groningen, University Medical Center Groningen, Groningen, The Netherlands

**Keywords:** Biomarker, cirrhosis, liver failure, microvasculature, plasmin, von Willebrand factor

## Abstract

**Background:**

In patients with acute and chronic liver disease, von Willebrand factor (VWF) antigen levels are markedly elevated, whereas a disintegrin and metalloproteinase with thrombospondin type 1 motifs, member 13 (ADAMTS13) activity is often reduced. The role of VWF proteolysis by other proteases, such as plasmin, remains unclear.

**Objectives:**

To investigate whether plasmin-mediated VWF cleavage occurs in patients with acute and chronic liver disease and to assess its association with VWF parameters, ADAMTS13 activity, and fibrinolytic markers.

**Methods:**

Plasma samples from 191 patients with stable or decompensated cirrhosis, acute liver failure or acute liver injury, acute-on-chronic liver failure, or sepsis without underlying chronic liver disease and 41 healthy controls were analyzed. VWF antigen and collagen-binding activity, ADAMTS13 antigen and activity, ADAMTS13-cleaved VWF, plasmin-cleaved VWF (cVWF), plasmin-α2-antiplasmin complexes, and D-dimer were measured by ELISA.

**Results:**

VWF antigen levels were higher in all patient groups and increased with disease severity. VWF activity was also elevated but was not proportional to VWF antigen level. ADAMTS13 activity and specific activity decreased with worsening disease. cVWF was undetectable in healthy controls and patients with stable cirrhosis but was increased in patients with decompensated cirrhosis, acute liver failure or injury, and acute-on-chronic liver failure. cVWF levels correlated with D-dimer but not with plasmin-α2-antiplasmin complexes or VWF activity.

**Conclusion:**

cVWF is detectable in patients with decompensated cirrhosis, acute liver failure or injury, and acute-on-chronic liver failure but not in those with stable cirrhosis or healthy individuals. Its association with D-dimer supports a link with fibrinolytic activation. These findings suggest that cVWF may reflect disease severity or ongoing microvascular thrombosis in patients with advanced liver disease.

## Essentials

• Plasma levels of von Willebrand factor (VWF) are markedly elevated in patients with liver disease.
• We assessed VWF cleavage by plasmin in patients with varying severity of liver disease.
• Plasmin-cleaved VWF was detectable in acutely ill patients, and levels correlated with D-dimer.
• Plasmin-cleaved VWF may reflect a response to ongoing microvascular thrombosis.

## 1 Introduction

Liver diseases are frequently associated with major alterations in the hemostatic system. Plasma levels of most hemostatic proteins are profoundly reduced in patients with worsening liver disease [1]. However, a few proteins that are synthesized by endothelial cells and not by hepatocytes may be elevated in these patients due to chronic endothelial activation. Indeed, von Willebrand factor (VWF) antigen levels are markedly elevated in patients with both acute liver injury and acute liver failure syndromes and in advanced chronic liver disease defined by underlying cirrhosis, and they increase with disease severity [2,3]. VWF has therefore been proposed as a noninvasive biomarker for disease progression and clinical outcomes, particularly in patients with advanced chronic liver disease [3]. However, the elevation in VWF antigen (VWF:Ag) is not matched by a proportional increase in VWF activity (VWF:Act) in patients with acute and chronic liver diseases. Previous studies have shown that the VWF:Act/VWF:Ag ratio decreases with worsening disease, suggesting functional impairment of circulating VWF [2]. This functional loss has been attributed to a reduction in high molecular weight (HMW) multimers, which are the most hemostatically active forms of VWF. Indeed, a reduction of HMW multimers may explain the decrease in activity-to-antigen ratio and the reduced binding to platelets and collagen observed in patients with acute and chronic liver diseases. However, the mechanism underlying HMW multimer loss remains unclear [2,4].

Plasma levels of a disintegrin and metalloproteinase with thrombospondin type 1 motifs, member 13 (ADAMTS13), the protease responsible for the physiological regulation of VWF multimer size, are significantly reduced in patients with acute and chronic liver diseases. While reduced ADAMTS13 levels impair processing of HMW multimers, the observed HMW multimer loss in patients with liver disease may also result from consumption due to formation of platelet-rich microthrombi in the liver or other tissues [2,[4], [5], [6], [7]]. In addition, proteolytic mechanisms other than ADAMTS13 may contribute to the loss of HMW multimers [8,9]. Plasmin, the central effector of fibrinolysis, has been implicated in VWF proteolysis in cirrhosis, based on the identification of plasmin-generated cleavage fragments in patient plasma [10].

A substantially skewed balance between elevated VWF:Ag levels and reduced ADAMTS13 activity may lead to the obstruction of microcirculation due to accumulation of HMW multimers, which subsequently triggers extensive local plasmin generation in an attempt to initiate a compensatory fibrinolytic response [11]. In this context, VWF can be cleaved by plasmin, resulting in the formation of a biochemically distinct, plasmin-specific cleavage product known as plasmin-cleaved VWF (cVWF) [12]. Although assays to detect ADAMTS13-specific cleavage products have been described previously, a bioassay to quantify cVWF in plasma has only recently become available [12]. Differentiating between ADAMTS13-mediated and plasmin-mediated VWF cleavage in patients with different severities of acute and chronic liver disease may offer mechanistic insight into VWF dysfunction and shed light on whether elevated cVWF levels reflect fibrinolytic activation in response to microvascular thrombosis.

In this study, we assessed VWF and ADAMTS13 parameters, cVWF levels, and markers of fibrinolytic activity in patients with acute and chronic liver disease across varying disease severities, as well as in patients with sepsis without underlying liver disease.

## 2 Methods

### 2.1 Study population

The study design, including detailed inclusion and exclusion criteria as well as patient characteristics, has been described previously by Elvers et al. [13]. In brief, citrated plasma samples were obtained from 191 patients with acute and chronic liver disease or sepsis without underlying liver disease admitted to King’s College Hospital (London, United Kingdom) between June 2017 and August 2021. Ethical approval was granted by the National Research Ethics Service Committee London - Westminster (Study Number 12/LO/1417) in accordance with the Declarations of Helsinki and Istanbul. Informed consent was obtained in writing from all participants or, in case of mental incapacity, from their personal consultees. Of the 191 patients, 168 had liver disease and were categorized into the following subgroups: stable cirrhosis (*n* = 39), decompensated cirrhosis (*n* = 63), acute-on-chronic liver failure (*n* = 50), and acute liver injury or acute liver failure (*n* = 16). In addition, 23 patients with sepsis without underlying chronic liver disease were included to distinguish the effects of liver dysfunction from systemic infection in critically ill patients, as previously described [14]. To establish reference values, plasma samples were also collected from 41 healthy volunteers at the same center.

### 2.2 Assays

VWF:Ag levels were measured using an in-house ELISA with commercially available polyclonal antibodies against VWF (DAKO). VWF:Act (collagen binding) was measured using a commercial ELISA kit (Zymutest VWF:CBA; Hyphen Biomed) according to the manufacturer’s instructions. ADAMTS13 activity was determined using the FRETS-VWF73 assay (Peptanova). To prevent interference from bilirubin, plasma samples were pretreated with bilirubin oxidase (2.5 U/mL; Sigma-Aldrich) for 30 minutes at 37 °C prior to analysis. Antigen and activity levels of VWF and ADAMTS13 in pooled normal plasma (a generous gift from Dr J.C.M. Meijers, Amsterdam University Medical Centers, the Netherlands) were defined as 100%, and results from test samples were expressed as a percentage of this reference. ADAMTS13 antigen levels were quantified using a commercially available ELISA kit (R&D Systems). ADAMTS13-cleaved VWF was measured by ELISA using a neoepitope-specific monoclonal antibody (MAB27642; R&D Systems), according to the method described by Rauch et al. [15]. cVWF levels were measured by ELISA using variable domain of heavy-chain-only antibodies as previously described by El Otmani et al. [12]. Plasma levels of plasmin-α2-antiplasmin (PAP) complexes were measured using a commercial ELISA kit (Technoclone). D-dimer was measured with the StaCompact 3 (Stago) using reagents and protocols provided by the manufacturer. Coefficients of variation of the various tests employed were <10%, with the exception of ADAMTS13 antigen and ADAMTS13-cleaved VWF levels, which were <15%.

### 2.3 Statistical analysis

Statistical analysis was performed using GraphPad Prism version 9.3.0 for Windows (GraphPad Software). Data are expressed as means. Normality was assessed using the Shapiro-Wilk test and the D’Agostino and Pearson test. Plasma analyte levels were compared between patient groups and reference values measured in healthy individuals using nonparametric one-way anova (Kruskal-Wallis test), followed by Dunn’s post hoc test. Correlations were assessed using linear regression with Spearman’s correlation coefficients. *P* ≤ .05 was considered statistically significant.

## 3 Results

### 3.1 Patient characteristics

We studied patients with stable cirrhosis (*n* = 39), decompensated cirrhosis (*n* = 63), acute-on-chronic liver failure (*n* = 50), and acute liver injury or acute liver failure (*n* = 16). In addition, we included patients with sepsis without underlying liver disease (*n* = 23) to dissect the effects of liver disease and infection in critically ill cirrhosis patients, as described previously. Patient values were compared with those of 41 healthy controls. Demographic, clinical, and laboratory characteristics of patients and controls are summarized in the Table [13].

**Table:** Demographic, clinical, and laboratory data of patients and controls.

Variable	Control (*n* = 41)	Stable cirrhosis (*n* = 39)	Decompensated cirrhosis (*n* = 63)	ACLF (*n* = 50)	ALI/ALF (*n* = 16)	Sepsis (*n* = 23)
Age, y	32.9 ± 7.8	57.1 ± 9.3	54.2 ± 11.9	50.2 ± 8.8	39.9 ± 13.2	59.4 ± 12.1
Male	14 (34)	29 (74)	45 (71)	39 (78)	10 (63)	15 (65)
Etiology						
Autoimmune hepatitis	-	0 (0.0)	0 (0.0)	1 (2)	3 (19)	-
Biliary	-	6 (15)	9 (14)	1 (2)	1 (6)	-
Budd-Chiari Syndrome	-	0 (0.0)	0 (0.0)	1 (2)	0 (0.0)	-
Cryptogenic	-	1 (3)	4 (6)	1 (2)	0 (0.0)	-
Drugs and toxins	-	17 (44)	38 (60)	37 (74)	3 (19)	-
Infection	-	8 (21)	2 (3)	1 (2)	0 (0.0)	-
MASLD	-	5 (13)	9 (14)	8 (16)	1 (6)	-
Sepsis without Liver disease	-	-	-	-	-	23 (100)
Others^a^	-	0 (0.0)	0 (0.0)	0 (0.0)	8 (50)	-
Wilsons’s disease or hemochromatosis	-	2 (5)	1 (2)	0 (0.0)	0 (0.0)	-
Examination						
BMI, kg/m^2^	22.5 ± 2.9	26.3 ± 4.0	27.2 ± 5.2	28.0 ± 5.4	27.1 ± 7.1	29.5 ± 8.1
Mean arterial pressure (calculated), mmHg	N/A	91.2 ± 11.2	81.8 ± 9.7	77.1 ± 11.2	83.4 ± 14.8	77.8 ± 10.9
Ascites grade 1 or 2 (mild to severe)	N/A	3 (8)	39 (62)	37 (74)	2 (13)	-
Hepatic encephalopathy grade 3-4	N/A	0	0	13 (27)	3 (21)	-
Child-Pugh scores	N/A	5.4 ± 0.5	9.4 ± 1.7	10.6 ± 2.1	-	-
MELD score	N/A	8.8 ± 1.6	20.2 ± 4.6	31.8 ± 7.3	-	-
Hematology and biochemistry on admission						
Alanine aminotransferase, IU/L	N/A	26 [10-105]	43 [11-401]	37.5 [10-371]	2035 [60-5511]	35 [17-1120]
Aspartate aminotransferase, IU/L	N/A	31 [6-109]	68 [22-581]	80 [12-1292]	629.5 [86-6369]	81 [13-3718]
Bilirubin (total), mg/dL	N/A	0.9 ± 0.5	4.8 ± 3.1	13.0 ± 12.5	13.3 ± 10.1	1.1 ± 1.0
Creatinine, mg/dL	N/A	0.8 [0.6-1.3]	0.8 [0.3-1.8]	1.3 [0.0-9.7]	1.3 [0.4-3.8]	1.1 [0.0-6.3]
Fibrinogen, g/L	N/A	3.5 ± 0.8	3.3 ± 3.6	2.3 ± 1.2	2.0 ± 1.5	4.8 ± 0.9
Hemoglobin, g/dL	N/A	134 [63-163]	103 [79-146]	85.5 [66-138]	106 [84-153]	92 [75-133]
INR	N/A	1.2 [1.0-1.5]	1.6 [1.1-2.4]	2.0 [1.1-4.7]	2.3 [1.0-7.7]	1.2 [1.0-6.2]
Na^+^, mmol/L	N/A	138.2 ± 3.7	133 ± 4.1	137.6 ± 7.4	139.5 ± 5.7	140.3 ± 5
Platelet count (×10^9^/L)	N/A	158 [45-359]	105 [23-253]	86 [20-271]	127.5 [27-837]	207 [52-669]

Mean ± SD or, as appropriate, median [IQR] are reported for continuous variables and count (percentage) for categorical variables.

BMI, body mass index; ACLF, acute-on-chronic liver failure; ALF/ALI, acute liver failure/injury; INR, international normalized ratio; MASLD, metabolic dysfunction-associated steatotic liver disease; MELD, model of end stage liver disease; N/A, not assessed.

This table was modified from Table published previously [13].

a Other etiologies for ALI/ALF included paracetamol overdose, hypotension, necrotizing pancreatitis, and rhabdomyolysis.

### 3.2 VWF:Ag and VWF:Act levels across disease severities

**Figure 1:**
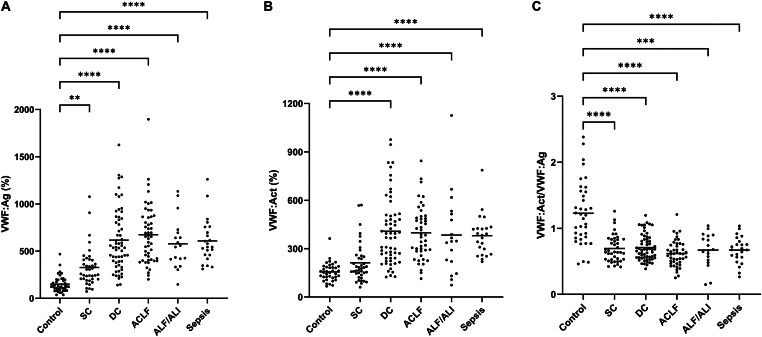
von Willebrand factor antigen (VWF:Ag) (A), VWF activity measured by collagen binding (VWF:Act) (B), and VWF activity-to-antigen ratio (VWF:Act/VWF:Ag) in patients with stable cirrhosis (SC) (*n* = 40), decompensated cirrhosis (DC) (*n* = 60), acute-on-chronic liver failure (ACLF) (*n* = 50), acute liver failure (ALF) or acute liver injury (ALI) (*n* = 18), and sepsis (*n* = 23) compared with healthy controls (*n* = 41). Horizontal lines indicate means. ∗∗*P* < .01, ∗∗∗*P* < .001, ∗∗∗∗*P* < .0001, all vs control.

Plasma VWF:Ag levels were quantified in patients and healthy controls to assess changes in total circulating VWF across disease stages. VWF:Ag was significantly elevated in all patient groups compared to healthy controls (Figure 1A). Median levels showed an overall increase with disease severity. In contrast, VWF:Act, measured by collagen binding, did not increase proportionally to antigen levels (Figure 1B), resulting in a decline in the VWF:Act/VWF:Ag ratio across disease stages (Figure 1C).

### 3.3 ADAMTS13 function is altered in decompensated and acute liver disease

**Figure 2:**
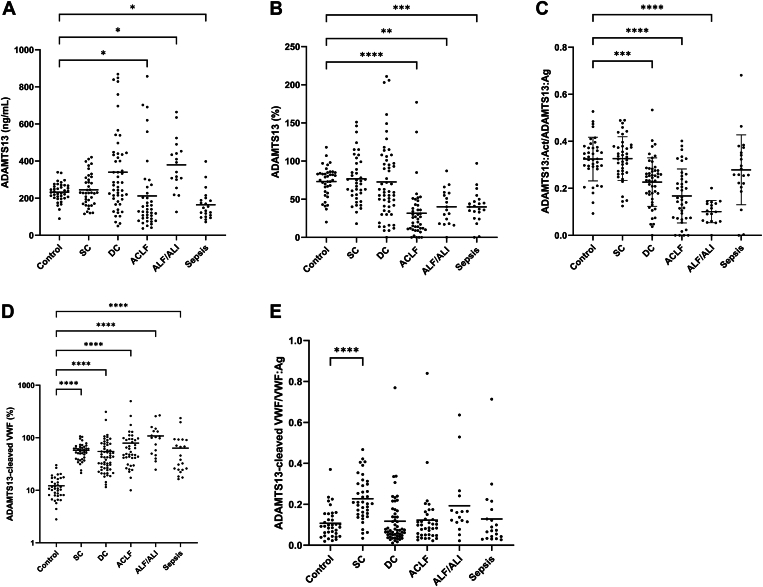
A disintegrin and metalloproteinase with thrombospondin type 1 motifs, member 13 (ADAMTS13) antigen (A), activity (B), specific activity (C), ADAMTS13-cleaved von Willebrand factor (VWF) (D), and ADAMTS13-cleaved VWF relative to VWF antigen (VWF:Ag) (E) in patients with stable cirrhosis (SC) (*n* = 40), decompensated cirrhosis (DC) (*n* = 60), acute-on-chronic liver failure (ACLF) (*n* = 50), acute liver failure (ALF) or acute liver injury (ALI) (*n* = 18), and sepsis (*n* = 23) compared with healthy controls (*n* = 41). Horizontal lines indicate means. ∗*P* < .05, ∗∗*P* < .01, ∗∗∗*P* < .001, ∗∗∗∗*P* < .0001, all vs control.

Given the disproportionate increase in VWF:Ag relative to VWF:Act levels, we investigated whether proteolytic cleavage by ADAMTS13 might explain the observed loss of function. Median ADAMTS13 antigen levels were comparable between patients and controls, except for higher values in patients with acute liver failure or acute liver injury and lower levels in those with acute-on-chronic liver failure and sepsis. Notably, the variability in ADAMTS13 antigen levels was much higher in patients than in controls, with levels in patients that were both higher and lower than the maximum and minimum values observed in controls (Figure 2A). In contrast, ADAMTS13 activity decreased in patients with acute-on-chronic liver failure, acute liver failure/acute liver injury, and sepsis (Figure 2B), resulting in a decline in ADAMTS13-specific activity proportional to disease severity (Figure 2C), indicating reduced enzymatic capacity in the most critically ill patients. Absolute levels of ADAMTS13-cleaved VWF were higher than controls in all patient groups (Figure 2D). However, when corrected for total VWF:Ag, ADAMTS13-cleaved VWF was increased in patients with stable cirrhosis but was comparable to controls in the other patient groups (Figure 2E).

### 3.4 Plasmin-mediated VWF cleavage increases with disease severity

**Figure 3:**
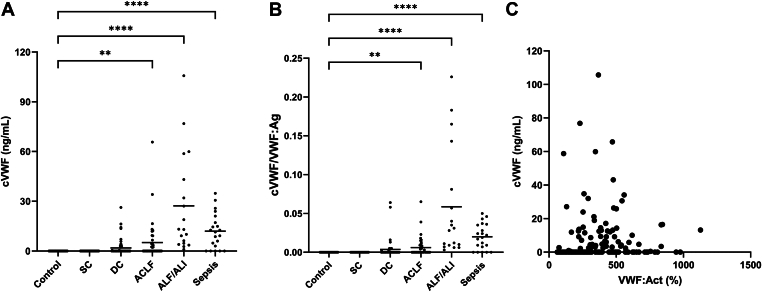
Plasmin-cleaved VWF (cVWF) measured in absolute concentrations (A), normalized to VWF antigen (VWF:Ag) (B), and correlated with VWF activity (VWF:Act) (C) in patients with stable cirrhosis (SC) (*n* = 40), decompensated cirrhosis (DC) (*n* = 60), acute-on-chronic liver failure (ACLF) (*n* = 50), acute liver failure (ALF) or acute liver injury (ALI) (*n* = 18), and sepsis (*n* = 23) compared with healthy controls (*n* = 41). Horizontal lines indicate means. ∗∗*P* < .01, ∗∗∗∗*P* < .0001, all vs control.

To evaluate plasmin-mediated proteolysis of VWF, we measured plasma levels of cVWF in absolute concentrations (Figure 3A) and normalized these values to total VWF:Ag (Figure 3B). In line with previous findings, cVWF was not detected in healthy controls [12]. Similarly, no measurable cVWF was observed in patients with stable cirrhosis. In contrast, cVWF became detectable in a subset of patients with more advanced cirrhosis, acute liver failure or acute liver injury, and sepsis with both the proportion of positive cases and cVWF levels increasing with disease severity. No significant correlation was observed between cVWF levels and VWF:Act, as measured by collagen-binding assay (Figure 3C).

### 3.5 Fibrinolytic markers diverge in advanced disease

**Figure 4:**
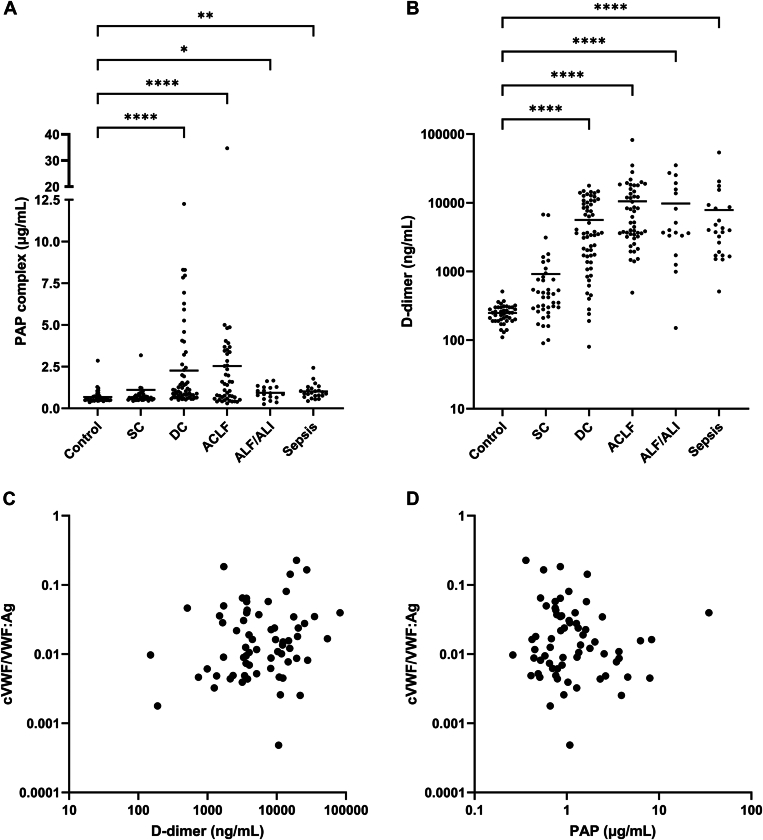
Plasmin-α2-antiplasmin (PAP) complexes (A) and D-dimer (B) in patients with stable cirrhosis (SC) (*n* = 40), decompensated cirrhosis (DC) (*n* = 60), acute-on-chronic liver failure (ACLF) (*n* = 50), acute liver failure (ALF) or acute liver injury (ALI) (*n* = 18), and sepsis (*n* = 23) compared with healthy controls (*n* = 41). Correlation between plasmin-cleaved VWF (cVWF) and D-dimer (C) and between cVWF and PAP complexes (D). Horizontal lines indicate means. ∗*P* < .05, ∗∗*P* < .01, ∗∗∗∗*P* < .0001, all vs control.

To assess whether plasmin-mediated VWF cleavage coincided with systemic activation of fibrinolysis, we measured plasma levels of PAP complexes and D-dimer. PAP complexes were significantly elevated in all patient groups compared to healthy controls, except in patients with stable cirrhosis (Figure 4A). D-dimer was significantly increased across all groups and increased progressively with disease severity (Figure 4B). D-dimer moderately correlated with cVWF:VWF:Ag (Spearman *r* = 0.47) (Figure 4C), whereas no significant correlation was observed between cVWF and PAP complexes (Figure 4D).

## 4 Discussion

Here, we confirm and extend previous findings on VWF functionality in patients with acute and chronic liver disease [2,[16], [17], [18], [19]]. In line with previous studies, we found elevated levels of VWF with decreased functional capacity in patients with chronic and acute liver disease. The decreased functional capacity appears to be related to plasmin-mediated proteolysis in some of the patients with very advanced disease (ie, decompensated cirrhosis/acute-on-chronic liver failure and acute liver failure or acute liver injury), whereas no cVWF was detected in patients with stable cirrhosis. Plasmin-mediated VWF proteolysis in patients with very advanced liver disease is accompanied by plasmin-mediated fibrin proteolysis as evidenced by the correlation between cVWF and D-dimer. Surprisingly, we did not find a correlation between cVWF and PAP complexes or cVWF and collagen-binding activity.

In addition to the previously identified reduced VWF functionality and VWF/ADAMTS13 imbalance, we have identified a profound decrease in ADAMTS13 functionality proportional to the severity of disease. We also observed individual patients with undetectable ADAMTS13 activity but detectable ADAMTS13 antigen, suggesting complete functional inactivation of the enzyme. Posttranslational modifications such as citrullination [20], arginine methylation [21], and oxidation [22] have been shown to reduce ADAMTS13 enzymatic activity. Enhanced formation of neutrophil extracellular traps [23] and oxidative stress [24] are hallmarks of liver disease and may explain the decreased specific activity of ADAMTS13. Moreover, enhanced protein arginine methylation may occur in patients with liver diseases and may therefore contribute to reduced ADAMTS13 activity [25]. Why acute liver failure or acute liver injury patients showed relatively preserved ADAMTS13-specific activity compared to patients with decompensated cirrhosis and acute-on-chronic liver failure is unclear but may reflect the fundamentally different pathological changes driving acute liver failure compared with hepatic decompensation in patients with cirrhosis. It may be that patients with chronic liver disease develop inhibitory antibodies against ADAMTS13, as has been described previously, whereas inhibitors do not develop in patients with acute liver failure or acute liver injury [26].

cVWF has been previously proposed as a biomarker for microvascular thrombosis, based on the concept that cVWF is only generated from thrombi formed in an environment of endothelial distress [12]. Previous studies in animal models and clinical observations in humans have demonstrated platelet- and VWF-containing microthrombus formation within the diseased liver [[27], [28], [29]]. However, whereas intrahepatic microthrombus formation is observed in essentially all animal models with liver injury, platelet and VWF deposition is only seen in a minority of biopsies from patients with advanced liver disease [29]. As we found detectable cVWF in only a minority of patients studied, cVWF may also serve as a marker of (intrahepatic) microthrombosis in patients with liver disease. Given that VWF-dependent intrahepatic microthrombosis appears to drive progression of disease [30], cVWF may help identify those patients at increased risk of poor outcomes. Adequately powered studies are required to assess the predictive value of cVWF. Such studies might be complemented by preclinical studies assessing the role of plasmin-mediated VWF proteolysis in clearance of intrahepatic microthrombi and progression of disease, for example, using targeted agents such as Microlyse, a fusion protein that combines the plasminogen activator urokinase with a VWF-targeting antibody [31].

An alternative scenario for the generation of cVWF in patients with acute and chronic liver disease is proteolysis of circulating VWF or VWF freshly released from endothelial cells by systemically or locally generated plasmin. Generation of cVWF by proteolysis of VWF not incorporated into thrombi would partially explain the reduction in HMW VWF multimers and associated decline in VWF functionality. In addition, the decreased functional capacity of VWF may relate to enhanced ADAMTS13-mediated VWF proteolysis and to VWF proteolysis by other enzymes including neutrophil-derived proteases such as elastase or cathepsin G [9]. A final mechanism underlying the decrease in HMW VWF multimers would be retention of these reactive multimers at the endothelial cell surface and/or consumption within microthrombi.

Our observation that cVWF levels correlate with D-dimer but not with PAP levels is surprising, as generation of plasmin is a prerequisite for the generation of all 3 biomarkers. The study of protein biomarkers in patients with liver disease, however, is invariably complicated due to the role of the liver in their clearance. The lack of correlation between cVWF and PAP levels may therefore relate to differential clearance kinetics. Of note, D-dimer and PAP complexes have substantially different half-lives, which may explain the divergence in correlation of cVWF with D-dimer and PAP levels [32]. Alternatively, the lack of correlation between PAP complexes and cVWF levels may suggest that α2-antiplasmin inhibition may not fully reflect plasmin activity in vivo. Other plasma protease inhibitors, such as α1-antitrypsin, α2-macroglobulin, and C1-inhibitor, can also bind and inactivate plasmin [33]. Among these, α1-antitrypsin is of particular interest, as its levels are often maintained or even increased in patients with liver disease [34]. This raises the possibility that alternative inhibitors may contribute to plasmin regulation, potentially limiting PAP complex formation despite ongoing plasmin-mediated VWF cleavage.

Although cVWF levels increased with disease severity, they did not correlate with VWF:Act as measured by collagen-binding assay, even after correction for total VWF:Ag. This suggests that cVWF may not substantially impair collagen-binding capacity or that the collagen-binding assay is not sensitive to the structural alterations induced by plasmin cleavage. Similar observations have been made in cirrhosis, where high collagen-binding activity was observed despite a relatively low ratio of collagen binding to total antigen, indicating that collagen-binding assays may overestimate VWF function when multimeric structure is altered [18].

This study amplifies the notion that VWF homeostasis in patients with acute and chronic liver disease is complex, with changes in multimeric size, unique cleavage products in a subset of patients, and functional defects in both VWF and ADAMTS13. Given the interest in VWF:Ag as a biomarker of disease severity and mortality, in-depth knowledge of the heterogeneity of VWF species circulating in patients with acute and chronic liver disease is key [3,35,36].

In conclusion, we found detectable levels of cVWF in patients with acute liver failure or acute liver injury and advanced chronic liver disease, except those with stable cirrhosis. We propose that plasmin-mediated VWF proteolysis may in part explain the decrease in functionality of circulating VWF, and that cVWF may signal intrahepatic microthrombosis in these patients. In addition, given the profound decrease in ADAMTS13 levels and specific activity, we surmise the existence of backup mechanisms to control VWF reactivity in patients with acute liver failure and advanced chronic liver disease as we and others previously proposed [11,37]. Follow-up studies to address the predictive value of cVWF in these patient populations are indicated, as are studies that treat VWF-dependent intrahepatic microthrombosis with the aim to halt disease progression.
